# Hsien-wen Wu: a founder of the studies of ichthyology in China

**DOI:** 10.1093/procel/pwac054

**Published:** 2022-12-05

**Authors:** Shunping He, Yiyu Chen

**Affiliations:** State Key Laboratory of Freshwater Ecology and Biotechnology, Institute of Hydrobiology, Chinese Academy of Sciences, Wuhan 430072, China; State Key Laboratory of Freshwater Ecology and Biotechnology, Institute of Hydrobiology, Chinese Academy of Sciences, Wuhan 430072, China

**Figure 1.**
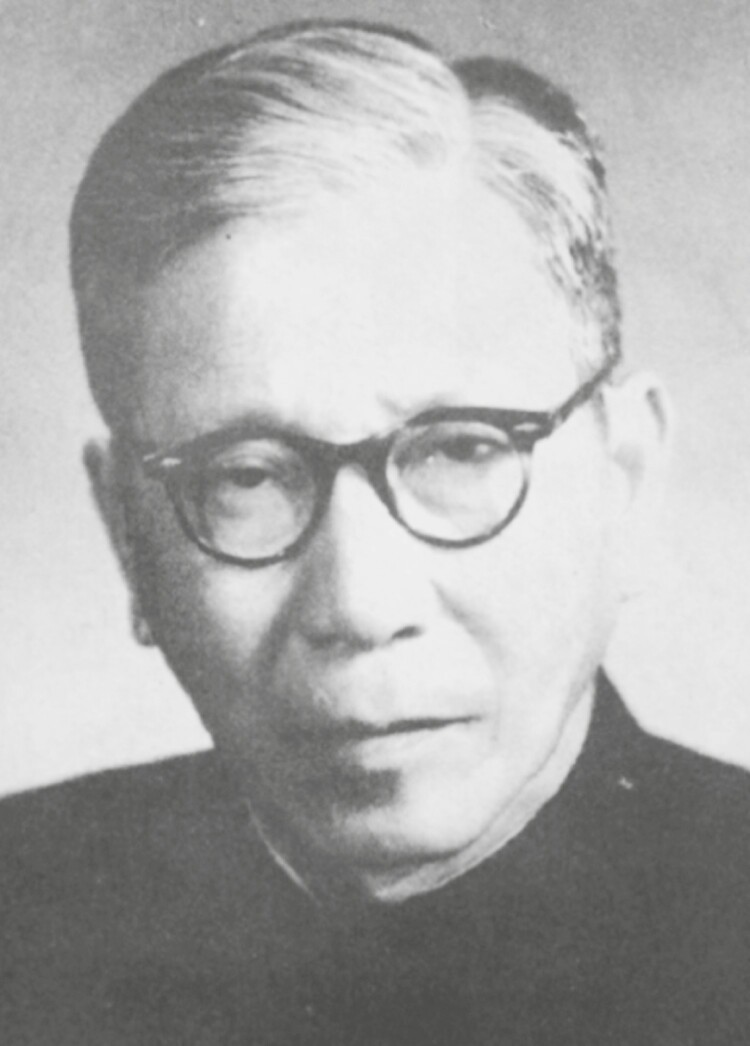
Dr. Hsien-wen Wu 1900–1985 (Chen, 1986).

The three-volume *Osteichthyes*, *Cypriniformes* in *Fauna Sinica* was finally completed in 2021. After the efforts of four generations of ichthyologists, the last volume has been officially submitted to the Science Press for printing. The publication of this book is a lifelong dream of the Academician Hsien-wen Wu (伍献文, H.W. Wu) (Fig. 1), a famous ichthyologist in China.

Hsien-wen Wu was born in the city of Ruian, Zhejiang Province, in 1900. After graduating from Xiamen (Amoy) University in Chen (1986), he joined the teaching staff in the Central University and began research work on fishes and their parasitic nemathelminths. His first ichthyological report, entitled “Study of fishes of Amoy,” was published in 1929. In 1932, he graduated from the National Museum of Natural History in Paris, obtained a Doctor of Science degree with his dissertation “Contribution a l’Etude, morphologique, biologique et systematique des poisons heterosomata (Pisces Heterosomata) de la Chine,” and was recommended to preside over the research work of the Institute of Zoology and Botany in *Academia Sinica*. In the same time, he held a position concurrently as a professor in zoology, anatomy, embryology, and parasitology in the Central University, Fudan University, and Jiangsu Medical College, respectively. His research objects were not only fish, but also included helminths, amphibians, and reptiles. During World War II, the Institute of Zoology and Botany moved from Nanjing to Chonqing, where Professor Wu devoted himself to studies on experimental ichthyology, including physiology and functional morphology such as the mechanism of aerial respiration in fish. From 1940 to 1947, Professor Wu guided his early students, Dr. Jiankang Liu (刘建康) and Dr. Xiaowei Zhang (张孝威), the famous ichthyologists in China, in serially publishing many scientific reports on the respiration mechanism of rice eel, exploring in relative detail the structure and function of respiration organ of rice eel based on the morphology, histology, and physiology. In 1950, the Institute of Zoology and Botany was renamed the Institute of Hydrobiology (IHB), Chinese Academy of Sciences (CAS), and moved from Shanghai to Wuhan. From 1964 to 1977, Dr. Wu devoted himself to the editing of the two-volume monograph *The Cyprinid Fishes of China* (including 113 genera, 412 species), and systematically gave a comprehensive description of freshwater fishes in China (Wu 1964, 1977). The publication of this book ended an era of consulting many ichthyological articles scattered in scientific journals in the past decades, and laid a good foundation for the later compilation of “*Fauna Sinica*, *Osteichthyes*, *Cypriniformes*” (Chen et al., 1998; Yue et al., 2000; Cao et al., 2022). Also, in 1953, one of the excellent students of Professor Wu, Xiaowei Zhang, organized a 5-year duration survey of the resource of mackerel fish in fishery outside Yangtai and nearby; this survey fills the gap in marine fish ecology in China.

**Figure 2.**
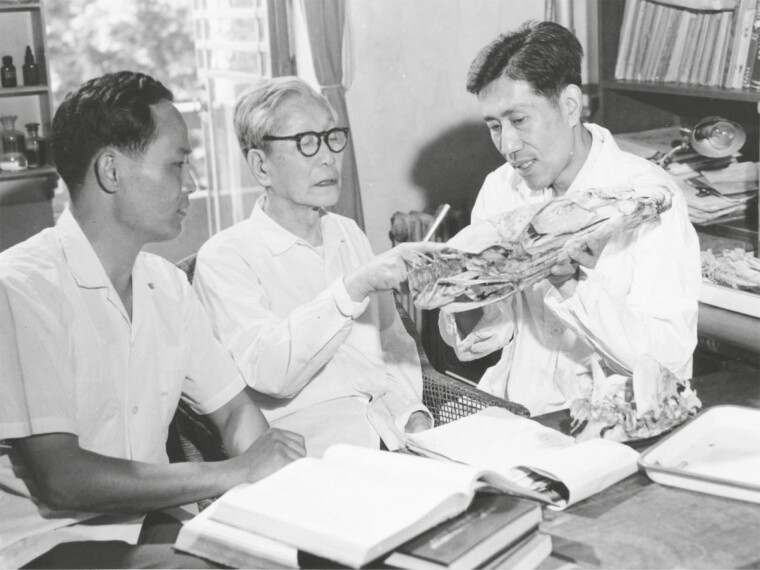
Dr. Hsien-wen Wu (in the middle) with the team members Dr. Yiyu Chen (front right) and Dr. Xianglin Chen (front left) in 1981.

Since “Systema Natura” was published by Carolus Linnaeus in 1758, binomial nomenclature has been widely applied in taxonomy. The binomial nomenclature has solved the name issue of species with the help of type specimens. However, the relationships among species and the level of classification were still an artificial empirical system, determined by maximum similarity at that time. The phylogenetic studies with ancestral and derived traits (the cladistic systematics) were not developed until 1970s. Since 1977, encouraged and promoted by Hsien-wen Wu, Yiyu Chen (陈宜瑜), Xianglin Chen (陈湘粦), and the members of the team have conducted an extensive skeletal study on the representative species in the order Cypriniformes, as well as the descending families such as Cyprinidae, Cobitidae, Homalopteridae, and Catostomidae (Fig. 2). They applied the cutting-edge cladistic systematic to analyze the evolutionary process of the Cyprinoidei, and finally published two important papers. One was entitled “Families division of the Cyprinidei and their systematic relationships” and was published in *Science in Chin*a in 1981 (Wu et al., 1981), and the other was entitled “Major groups within the family Cyprinidae and their phylogenetic relationships” and was published in *Acta Zoologica Sinica* in 1984 (Chen et al., 1984). These papers have expounded on the phylogenetic relationship of Cyprinid fish and successfully established the natural classification system of cypriniform fishes. The publication of these papers attracted extensive attention from the international zoological and ichthyological communities. British ichthyologist invited Dr. Yiyu Chen to conduct cooperative research in the British Museum. Professor Fink & Fink from the University of Michigan spoke highly of the work of Hsien-wen Wu’s team and wrote a long letter to discuss the scientific topics. The work of Hsien-wen Wu’s team has laid a solid foundation for the rapid development of ichthyology research in China, and these works have been included in the second edition of “Fishes of the World” and have been applied in the fish classification system at that time (Nelson, 1984).

Professor Hsien-wen Wu paid high attention to the collection of fish specimens and emphasized the importance of long-term fieldwork. The team members Yiyu Chen and Wenxuan Cao (曹文宣) have long devoted themselves to field research on the Qinghai–Tibetan Plateau to collect specimens. Due to their efforts, China currently has the largest collection of cypriniform specimens in the world, and IHB has been rewarded to be one of the world’s leading centers for ichthyology, along with fish research centers in France, Sweden, the United Kingdom, and the United States. Until now, the fish museum founded by Hsien-wen Wu has collected more than 400,000 fish specimens, accounting for more than 1,200 species of fish.

Dr. Hsien-wen Wu emphasized the importance of frontier scientific research, which promoted the rapid development of the ichthyology research team in IHB. Following his heritage, the team has conducted a series of cutting-edge studies, including the evolution of Cyprinidae, Cypriniformes (He et al., 2004b, 2008b; Wang et al., 2006; Jian et al., 2020), and plateau fishes (Cao et al., 1981; Wu, 1984; He et al., 2004a; He and Chen, 2007; Yang et al., 2021), as well as the adaptation of abyssal fish (Shen et al., 2017; Wang et al., 2019). Due to the solid foundation built by Hsien-wen Wu and the good inheritance by Dr. Yiyu Chen et al., the research work of ichthyology in China has gradually kept pace with the times. With the further application of molecular systematic, we have stepped out of the mire of morphological convergence and maximum similarity, and corrected many mistakes in the taxonomy of Cypriniformes and Cyprinidae. Now we have suggested an independent origination of the East Asian Cyprinids under the East Asian monsoon climate and reconstructed the phylogeny of Cypriniformes. A large number of papers related to the molecular systematics of Cyprinidae and Cypriniformes have been published in *Molecular Phylogenetics and Evolution* (Wang et al., 2006; He et al., 2008a, 2008b; Li et al., 2008a; Duan et al., 2009; Zhao et al., 2011; Tao et al., 2013; Yang et al., 2015), *Science China* (He et al., 2004b; Zhao et al., 2005; Wang et al., 2016; Tao et al., 2019), *Chinese Science Bulletin* (He et al., 2001, 2004a, 2007; Li et al., 2008b), *Progress in Natural Science* (Wang et al., 2004; Li et al., 2005), and so on, which has been widely accepted by the international ichthyologist community and has been cited in the famous fifth edition of “Fishes of the World” (Nelson et al., 2016).

In summary, Hsien-wen Wu’s great contribution to ichthyological research in China is mainly manifested in the concentrated description of Cyprinidae and Cypriniformes, the publication of “The Cyprinid Fishes of China,” “*Fauna Sinica, Osteichthyes*, *Cypriniformes*,” and the launch of the phylogenetic studies on Cyprinidae and Cypriniformes.

Hsien-wen Wu’s great contribution also includes the establishment of the largest fish museum in Asia featuring Cypriniformes. After decades of improvement, there are now more than 400,000 fish specimens, about 1,200 species, and more than 260 type specimens housed in the museum.
